# Developing a Multisensor-Based Machine Learning Technology (Aidar Decompensation Index) for Real-Time Automated Detection of Post–COVID-19 Condition: Protocol for an Observational Study

**DOI:** 10.2196/54993

**Published:** 2025-03-27

**Authors:** Jenny Mathew, Jaclyn A Pagliaro, Sathyanarayanan Elumalai, Lauren K Wash, Ka Ly, Alison J Leibowitz, Varsha G Vimalananda

**Affiliations:** 1 Aidar Health, Inc Columbia, MD United States; 2 Center for Healthcare Organization and Implementation Research (CHOIR) VA Bedford Healthcare System Bedford, MA United States; 3 Clinical Informatics Providence VA Medical Center Providence, RI United States; 4 Boston University Chobanian & Avedisian School of Medicine Boston, MA United States

**Keywords:** Aidar Decompensation Index, AIDI, biophysical biomarkers of worsening health, biosensor-based physiological monitoring, cardiorespiratory, metabolic, renal, and neurological complications after COVID-19, early warning signs of clinical decompensation, long COVID, noninvasive monitoring of physiology, postacute sequelae of COVID-19, PACS, rapid assessment tool, risk triaging related to long COVID

## Abstract

**Background:**

Post–COVID-19 condition is emerging as a new epidemic, characterized by the persistence of COVID-19 symptoms beyond 3 months, and is anticipated to substantially alter the lives of millions of people globally. Patients with severe episodes of COVID-19 are significantly more likely to be hospitalized in the following months. The pathophysiological mechanisms for delayed complications are still poorly understood, with a dissociation seen between ongoing symptoms and objective measures of cardiopulmonary health. COVID-19 is anticipated to alter the long-term trajectory of many chronic cardiovascular and pulmonary diseases, which are common among those at risk of severe disease.

**Objective:**

This study aims to use a single, integrated device—MouthLab, which measures 10 vital health parameters in 60 seconds—and a cloud-based proprietary analytics engine to develop and validate the Aidar Decompensation Index (AIDI), to predict decompensation in health among patients who previously had severe COVID-19.

**Methods:**

Overall, 200 participants will be enrolled. Inclusion criteria are patients in the US Department of Veterans Affairs health care system; “severe” COVID-19 infection during the acute phase, defined as requiring hospitalization, within 3-6 months before enrollment; aged ≥18 years; and having 1 of 6 prespecified chronic conditions. All participants will be instructed to use the MouthLab device to capture daily physiological data and complete monthly symptom surveys. Structured data collection tables will be developed to extract the clinical characteristics of those who experience decompensation events (DEs). The performance of the AIDI will depend on the magnitude of difference in physiological signals between those experiencing DEs and those who do not, as well as the time until a DE (ie, the closer to the event, the easier the prediction). Information about demographics, symptoms (Medical Research Council Dyspnea Scale and Post-COVID-19 Functional Status Scale), comorbidities, and other clinical characteristics will be tagged and added to the biomarker data. The resultant predicted probability of decompensation will be translated into the AIDI, where there will be a linear relationship between the risk score and the AIDI. To improve prediction accuracy, data may be stratified based on biological sex, race, ethnicity, or underlying clinical characteristics into subgroups to determine if there are differences in performance and detection lead times. Using appropriate algorithmic techniques, the study expects the model to have a sensitivity of >80% and a positive predicted value of >70%.

**Results:**

Recruitment began in January 2023, and at the time of manuscript submission, 204 patients have been enrolled. Publication of the complete results and data from the study is expected in 2025.

**Conclusions:**

The focus on identifying predictor variables using a combination of biosensor-derived physiological features should enable the capture of heterogeneous characteristics of complications related to post–COVID-19 condition across diverse populations.

**Trial Registration:**

ClinicalTrials.gov NCT05220306; https://clinicaltrials.gov/study/NCT05220306

## Introduction

Severe complications of postacute COVID-19 infection like thrombosis, respiratory failure, and cardiac and vascular damage increase the likelihood of future morbidity and mortality in recovered patients. In one study of individuals who had been hospitalized with COVID-19, nearly a third (14,060/47,780, 29.4%) of individuals who were discharged from the hospital after acute COVID-19 were readmitted, and more than 1 in 10 (5875/47,780, 12.3%) died over a follow-up period of 140 days after discharge [1]. These events occurred at rates 4 and 8 times greater, respectively, than in the matched control group from the general population. In another study, the 6-month incidence of a new hospitalization among patients previously hospitalized with COVID-19 was found to be 29.8% [2]. SARS-CoV-2 infection can also lead to postacute development of substantial cardiac cellular abnormalities and cardiovascular clinical sequelae, including dysrhythmias, ischemic heart disease, heart failure, pericarditis, myocarditis, and thromboembolic disease [3]. Glycometabolic abnormalities are also evident in many survivors of acute COVID-19 and, in some cases, manifest as overt new-onset diabetes mellitus [4].

Risks of complications are higher among patients with comorbidities like congestive heart failure, chronic obstructive pulmonary disease, asthma, chronic kidney disease, diabetes, and hypertension [1,5,6]. Thus, although the current rate of new COVID-19 infection has dropped, the risk of morbidity, mortality, and organ dysfunction among the survivors of COVID-19 infection is high, especially among those with preexisting illnesses. Compelling evidence suggests that the pandemic may lead to new-onset kidney disease (and other noncommunicable chronic diseases including diabetes, cardiovascular disease, and neurological disease) in the millions. Given the scale and the chronic nature of several of its sequelae, post–COVID-19 condition (also known as “long COVID” or “postacute sequelae of COVID-19”) will reverberate with us for decades and will have broad and deep social and economic impact long after the COVID-19 pandemic abates [7]. Thus, it would be appropriate to infer that the next wave related to COVID-19 may not necessarily be a new strain but rather the surge of hospitalizations due to postacute complications. Developing strategies to prevent decompensation among high-risk survivors of COVID-19 has emerged as an unmet need.

One approach to the prevention of decompensation is to monitor for worsening in physiological parameters after hospital discharge. However, collecting such data longitudinally for prediction poses several challenges. First, only a limited number of parameters can be easily collected (eg, blood pressure, heart rate, oxygen saturation, etc), although additional parameters such as heart rate variability and lung function may provide additional prognostic data. Second, even if patients or families are able to collect these parameters longitudinally, there is no automated, validated method to analyze those data concurrently and provide a quantitative assessment of decompensation risk over time. These limitations hinder the sensitivity and thus the utility of current approaches to using physiologic parameters to predict decompensation risk among patients after COVID-19.

The overall objective of this study (trial registration: ClinicalTrials.gov NCT05220306) is to use a handheld, multisensor device—MouthLab, which measures 10 vital health parameters in 60 seconds—and a cloud-based proprietary engine to develop and validate the Aidar Decompensation Prediction Index (AIDI). MouthLab measures oral temperature, single-lead electrocardiogram, heart rate, breathing rate, heart rate variability, respiratory flow morphology, oxygen saturation, pulse rate, and basic lung function (forced expiratory volume in the first second and peak expiratory flow rate). The study will collect MouthLab data from patients previously hospitalized with COVID-19 (“severe COVID-19”) in order to identify decompensation predictor variables. These variables will be used to develop the AIDI to accurately predict decompensation events (DEs) related to post–COVID-19 condition. Index development will follow best practices in machine learning and algorithmic development, where the main outcome is any COVID-19–related event that leads to an emergency department (ED) visit, hospitalization, or the need for escalated care (new diagnosis or change in intervention to manage the chronic conditions). These DE cases will help inform the final set of predictor variables to develop the AIDI. In the future, early prediction and real-time risk triaging using the AIDI may support better clinical decision-making, thus preventing complications, controlling disease progression, and improving outcomes. The aim of this paper is to describe the protocol for this study to develop and validate the AIDI.

## Methods

### Study Design

This is a national, longitudinal, observational study. Potentially eligible patients will be identified using the US Department of Veteran Affairs (VA) Corporate Data Warehouse (CDW) and the COVID-19 Shared Data Resource. A total of 200 patients who have a history of “severe” COVID-19 infection in the previous 3-6 months and at least 1 of the specified comorbidities will be recruited. Severe COVID-19 is defined as requiring hospitalization for acute COVID-19 or its complications. Participants will be required to use the MouthLab device twice daily and to complete monthly surveys administered remotely either by phone or through a web portal. Data will be used to develop a machine learning–based algorithm to detect DEs among patients in the postacute COVID-19 phase.

### Study Setting

The VA is the largest integrated health system in the United States, with over 9 million patients enrolled. VA delivers care to US military veterans across 171 VA Medical Centers and 1113 outpatient care sites across the United States and Puerto Rico, Guam, and other US territories [8]. Nearly 50% of enrolled veterans are aged 65 years or older; 91.1% are male and 8.9% are female; and overall, veterans bear a greater burden of physical and mental health comorbidities than nonveterans [9,10].

### Sample Size

To develop the AIDI algorithm, the study will collect longitudinal parameters that lead up to a DE with the MouthLab device. Because the actual multiparametric changes are unknown and are, in fact, the outcome of the study, the study aims to obtain as much data as possible from those who experience a DE. For this study, we estimate that 100 individual DEs would be sufficient to create a statistical algorithm to predict decompensation. The ultimate goal is to have the algorithm produce no false negatives (ie, if someone is at risk of decompensation while the algorithm says that they are not) and less than 10% false positives (ie, if someone is *not* at risk of decompensation while the algorithm says they are). That is, we do not want to miss any DEs at the cost of having some nonevents classified as being risky.

Al-Aly Z et al [11] observed that the risk and associated burden of pulmonary and extrapulmonary manifestations increase across the disease spectrum of acute COVID-19 infection (from nonhospitalized individuals to hospitalized individuals and then to those admitted to intensive care). The study implemented a comparative approach to examining postacute sequelae in individuals who are hospitalized with COVID-19 versus individuals with seasonal influenza (using a high-dimensional approach and through examination of prespecified outcomes). Results suggested that there is a substantially higher burden of a broad array of postacute sequelae in the individuals who are hospitalized with COVID-19, which provides features that differentiate post–COVID-19 condition (both in the magnitude of risk and the breadth of organ involvement) from postinfluenza viral syndrome [11]. A study by Mainous et al [2] demonstrated a hospitalization rate of 29.8% among survivors of “severe COVID-19.” Given that the AIDI study has defined “decompensation” more broadly than hospitalization alone, we anticipate higher decompensation rates among our study population. Conservatively estimating 1 DE per any participant, with an event rate of 60%, we would need 168 participants to complete the study in order to capture 100 DEs. Since we anticipate up to 15% dropout, we will aim to recruit 200 participants.

### Eligibility Criteria

The inclusion and exclusion criteria are shown in Textbox 1.

**Inclusion and exclusion criteria.**

**Inclusion criteria**

- Aged 18 years and older
- Were hospitalized for treatment of acute COVID-19 or its complications in the prior 3-6 months
- At least one of the following comorbidities:
  Hypertension
  Asthma
  Chronic obstructive pulmonary disease
  Heart failure
  Chronic kidney disease
  Diabetes mellitus
- English fluency
- Comfortability with using technology
- Basic literacy with electronic platforms

**Exclusion criteria**

- Any motor disability that would impede the use of the MouthLab device as required with the left hand
- Pregnancy
- Cognitive deficits that would impede the ability to provide informed consent
- Alzheimer disease or other dementia
- Pacemakers and implantable cardioverter-defibrillators

### Recruitment

This study will recruit participants from across the country who receive care at the VA. A waiver of the Health Insurance Portability and Accountability Act (HIPAA) and informed consent for screening will be obtained for recruitment. Three modes of recruitment will be used. First, eligible patients will be identified via the CDW and sent letters with information about the study and a number to call if they are interested in learning more. Second, those who have authorized the VA through any partnership programs (eg, COVID-19 research volunteer registry) to receive communication on research opportunities may be contacted for this study. Third, study flyers will be posted at three of the VA’s post–COVID-19 condition clinics. The selection process will include best efforts to ensure equitable inclusion of different races, ethnicities, and age groups among the participants.

### Ethical Considerations

The protocol was approved by the Central Institutional Review Board of the Veterans Healthcare Administration, on December 21, 2022 (1720625-1). Since this is a multisite study, individual institutional review boards at the VA Bedford Healthcare System and the VA Providence Healthcare System also reviewed and approved the study. Informed consent will be obtained from all eligible participants prior to shipping the MouthLab device, administering baseline surveys, and collecting any patient data. Upon obtaining consent, the research team will also obtain HIPAA authorization. No study-related tasks will be performed before obtaining both consent and HIPAA authorization. A member of the research team will send the informed consent form (ICF) to the patient via DocuSign and read through the form with the participants on the WebEx (Cisco) consent call. The person obtaining consent will review the ICF in depth and solicit all questions before the participants sign the ICF via DocuSign. Participants will receive an executed copy via email, and those who prefer to sign a physical copy will be mailed a consent form. This approach will allow participants sufficient time to follow up with questions and discuss before officially signing the consent form. Participants will receive US $85 at the end of months 1-5. At the end of month 6, upon the completion of study tasks (survey and device return), participants will receive US $175. The total potential compensation is US $600, paid by check.

### Study Timeline

The timeline of the study is provided in Table 1.

In addition to MouthLab and survey data (Table 2), data will be collected from the CDW on patient demographics, comorbidities, medications, and potential DEs. The main DEs of interest are any COVID-19–related events that lead to an ED visit, hospitalization, or the need for escalated care (new diagnosis or change in intervention to manage the chronic conditions).

The study will collect information about the index hospitalization, related to COVID-19 either as a primary or secondary diagnosis, from the CDW. This information will include medications (including oxygen therapy), vitals, and the use of mechanical ventilation. Additionally, the index hospitalization will be classified as an intensive care unit–level admission or not.

**Table 1:** Timeline of patient data collection for the Aidar Decompensation Index study.

	Phone screening	Consent	Patient intake form	Training on the MouthLab device	MRC^a^ Dyspnea Scale [12]	Post-COVID-19 Functional Status Scale [13]	VR-12^b^ [14]	User experience survey
First contact and screening	✓							
Consenting and HIPAA^c^ authorization		✓						
Baseline and BP^d^ calibration				✓				
Monthly follow-ups and data collection			✓^e^		✓^f^	✓^f^	✓^e,g^	✓^g^
Termination								

^a^MRC: Medical Research Council.

^b^VR-12: Veterans RAND 12-Item Health Survey.

^c^HIPAA: Health Insurance Portability and Accountability Act.

^d^BP: blood pressure.

^e^First month: patient intake form and first VR-12.

^f^Monthly: MRC Dyspnea Scale and Post COVID-19 Functional Status Scale.

^g^Sixth month: second VR-12 and user experience survey.

**Table 2:** Survey data to be collected for Aidar Decompensation Index study.

Survey data	Instrument or measure	Time points
MRC^a^ Dyspnea Scale (score 1-5)	A 5-point scale that has been used to classify dyspnea, mainly used in grading COPD^b^ progression	Every 4 weeks
Post-COVID-19 Functional Status Scale (0-4 grade scale)	Functional status questionnaire that measures the impact of post–COVID-19 condition on ADL^c^ along with psychological symptoms	Every 4 weeks
VR-12^d^ Quality of Life Assessment	Patient self-report	At the start and end of study
MouthLab Usability Survey	MCQs^e^ to ascertain the information of the physical and usage aspects of the MouthLab device	Study conclusion
User feedback on remote physiological monitoring technology	General perception about remote monitoring technologies (wearable and spot-check)	Study conclusion
Index hospitalization	Collected laboratory data, diagnoses, investigation report, medications, oxygen therapy, clinical notes, and vital signs data	Study conclusion

^a^MRC: Medical Research Council.

^b^COPD: chronic obstructive pulmonary disease.

^c^ADL: activities of daily living.

^d^VR-12: Veterans RAND 12-Item Health Survey.

^e^MCQ: multiple-choice question.

### Evaluation of DEs

Data on outcomes (DEs) will be collected at the end of the data collection period via the CDW, records obtained from non-VA hospitalizations and ED visits, and monthly and end-of-study surveys.

A DE is defined as a complication related to organ dysfunction induced by SARS-CoV-19 that establishes the need for a change in treatment or intervention. To identify these events, we will first identify all new diagnoses and treatments, including medications, health care visits, and changes in level of service (eg, outpatient, inpatient, and hospital at home), for each patient during the study period using national CDW data. The data reviewed will be limited to those linked to relevant service types (eg, emergency care, urgent care, primary care, cardiology, and pulmonology). A study staff member will review these outcomes with guidance from the principal investigator or other team member with medical training to establish which are highly likely, somewhat likely, and not likely to be related to COVID-19. The full set of outcomes (Table 3) will be used as one outcome in one approach to the development of the predictive algorithm, and the subsets of highly likely and somewhat likely DEs will be used in a second approach to development.

**Table 3:** Study outcomes.

Outcomes		Instrument or measure
**Primary outcomes**		
	All-cause, higher-level health care use (hospitalization, ED^a^, or urgent care visits within the VA^b^ system)	Participant self-reporting, confirmed through EMRs^c^
	All-cause health care use (within and outside the VA system)	Participant self-reporting, confirmed through record requests and EMRs
	Highly likely COVID-19–related health care use	As evaluated by the PI^d^ and subinvestigators
	Highly likely and somewhat likely COVID-19–related health care use	As evaluated by the PI and subinvestigators
**Secondary outcomes**		
	Highly likely and somewhat likely COVID-19–related health care use	As evaluated by the PI and subinvestigators
	Highly likely COVID-19–related diagnoses, treatment, use, and medication	As evaluated by the PI and subinvestigators and confirmed in EMRs

^a^ED: emergency department.

^b^VA: Department of Veteran Affairs.

^c^EMR: electronic medical record.

^d^PI: principal investigator.

### Data Blinding

Participants and physicians are blinded to the data from the MouthLab device. Participants will be informed that they are not restricted from seeking any level of care. There will be no medical intervention provided during or after the study as a consequence of any unfavorable trends in the MouthLab data, as these data are not being surveilled during the study.

### Participant Data Protection

The confidentiality of participants will be maintained throughout the study. All participants will be assigned a unique subject ID by the data analyst. The crosswalk that matches the participant ID to the participant’s name will be secured behind the VA firewall. Any data that may be published in abstracts and scientific journals or presented at medical meetings will reference the unique participant ID or will be aggregated, so to not reveal the participant’s identity. Patient identifiable information such as name, address, email address, and phone number will only be used to support device shipment and return–related activities. Participants’ unique identifiers, rather than personal information, will be connected to their device and survey data as well as any other data obtained from the electronic medical record. To protect participants’ privacy, the Aidar Connect dashboard will not collect any patient identifiable information during registration, and pairing of MouthLab devices will be performed against the participants’ IDs.

### Safety Monitoring

During the monthly health surveys sent via the VA Office of Research and Development Qualtrics platform, participants will report any potential adverse events (AEs) that are possibly related to their COVID-19 diagnosis, as well as to any hospitalizations or medical care use. Study coordinators will follow up with participants to gather more information and have participants sign a Release of Information if needed. The investigator or study coordinator will determine whether the AE or serious AE meets the criteria for reporting; submit to the sponsor and VA Central Institutional Review Board all associated information and documentation related to each AE or SAE; and execute corrective actions as necessary.

### Statistical Analysis

#### Feature Engineering and Selection

For each patient, baseline readings will be based on their first few MouthLab measurements. The number of observations used to establish the baseline will depend on the variability in each patient’s reading. We will then summarize the patient’s parameters obtained from the MouthLab data collection. At each time t, we will include the most recent observations, changes since the last reading, trends, variability in values, and comparison to the patient’s baseline. Additionally, we will include information about demographics, symptom scores, other clinical characteristics, as well as key comorbidities. We anticipate that variable interactions will be important. Given the cohort size and the number of different features, regularization will be applied to avoid overfitting.

#### Outcome Modeling

We will define the outcome as patients experiencing a DE within τ time units (DEτ). For different values of τ, we will deploy a series of more sophisticated models in order to explore the predictive ability of different approaches, as detailed below. We will split up the data into training and testing datasets based on the time of enrollment. We anticipate an 80:20 split; in other words, we will set aside 20% of the data to evaluate the model performance. For each τ, we will construct a training dataset consisting of multiple overlapping time segments for each patient, which we refer to as the observation period (eg, 2 weeks), and multiple nonoverlapping time segments leading up to the outcome for the patients that experience the outcome, one of which contains the outcome.

Second, we will explore several other machine learning algorithms, including classification trees, random forest, and boosting approaches, to understand whether these more flexible modeling approaches have an advantage over the logistic regression approach. The output of any of these approaches is a predicted probability of a DE occurring within the following τ days given observations of a fixed observation interval (eg, up to 30 previous measurements, or ~2 weeks). As stated earlier, the goal is to develop the AIDI to predict worsening health. The detailed analysis laid out above will allow us to understand the ability of early prediction, that is, the critical time frame just prior to decompensation during which the probability of hospitalization becomes increasingly predictive.

The initial exploratory analysis will inform the selection of proper features for the analysis. We will model the patient’s risk of hospitalization as a function of their physiological values, short-term changes in those values, changes from the baseline, etc. The initial exploratory analysis will inform features such as the appropriate time ranges (eg, should short-term changes be captured since the last reading or over the last 24 hours).

#### Performance Evaluation

In case of a lower-than-expected number of observed outcomes, or smaller differences than expected between those who experience the outcome and those who do not, we may revert from our 80:20 split to use cross-validation to measure performance. In that case, we will evaluate the algorithm using the leave-one-out cross-validation technique. We will implement this approach by leaving out one patient at a time (corresponding to potentially multiple observation periods to avoid fitting the model on data from the same patient as the model is being evaluated). The leave-one-out cross-validation technique facilitates cross-validation evaluation of the algorithm structure to assess its robustness to changes. This validation step will remake the algorithm for every patient left out of the algorithm development iteration. In short, this approach fits each algorithm using all available data except for (in our case) the data of one patient and then summarizes whether the algorithm predicts the correct classification of decompensation or nondecompensation for each observation period. This process is repeated over all available patients. For each τ, we will summarize the accuracy, false positives, and false negatives.

The resulting predicted probabilities of decompensation will then be translated into the easy-to-communicate AIDI, where there is a linear relationship between the risk score and the AIDI. At the conclusion of phase 3, the final COVID-19 DE detection performance of the AIDI will be evaluated using standard measures including sensitivity, specificity, and positive predictive value across a wide range of risk cutoff values. Overall, AIDI DE detection performance and lead time statistics will be examined across study populations as well as within subgroups. If necessary, to improve prediction accuracy, data may be stratified based on biological sex, race, ethnicity, or underlying clinical characteristics into subgroups to determine if there are differences in performance and detection lead times among the groups.

## Results

Recruitment began in January 2023, and the first patient was enrolled in January 2023. At the time of manuscript submission, 204 patients have been enrolled. Publication of the complete results and data from the study is expected in 2025.

## Discussion

### Expected Findings

The AIDI study seeks to address a substantial challenge, that is, to develop an affordable, scalable, technology-assisted remote monitoring solution to facilitate early identification of abnormalities and timely intervention by clinicians to improve quality of life, functional status, and health outcomes and, ultimately, to reduce the burden of post–COVID-19 condition in the United States and elsewhere. We hypothesize that monitoring participants for 6 months and capturing critical cardiorespiratory parameters on a daily basis in conjunction with subjective and clinical data will enable the development of an index, the AIDI, that will empower high-quality clinical care using the MouthLab device as a rapid and early assessment tool. The outcomes of this research have the potential to redefine the care delivery for COVID-19 and potentially other conditions in the future, as well as push the boundaries of prevention and care beyond traditional care settings into the home.

### Limitations

Self-selection bias may be a limitation of this study as participation is voluntary, and participants already comfortable with using technology might be especially motivated to participate in digital intervention studies. Patients who are particularly ill may not enroll due to difficulty managing the device, and those without cellular service will not be able to participate.

### Strengths

Fully remote study procedures will enhance the accessibility of eligible research participants living in rural or remote locations away from large metropolitan areas. The MouthLab device has built-in SIM cards offering cellular data, thus requiring no other accessory, such as mobile phones, to transmit data to the Aidar Cloud. Using a web application dashboard will allow easier visualization of MouthLab data from all participants, providing us with the opportunity to intervene immediately in the event of absent data or noncompliance.

### Conclusions and Future Work

The findings from this study will result in a predictive algorithm (AIDI) to predict decompensation among high-risk patients who previously had severe COVID-19. In the short term, this could be applied to monitoring such patients during the hospital-to-home transition or for longer periods at home. In the long term, we will adapt and validate the AIDI for monitoring other conditions. Our work will generate insight and guidance for scalable and easy-to-use digital monitoring solutions for remote management of chronic diseases to control disease progression, limiting the impact of comorbidities and, ultimately, improving health outcomes among high-risk populations.

## Abbreviations

AE: adverse event
AIDI: Aidar Decompensation Index
CDW: Corporate Data Warehouse
DE: decompensation event
ED: emergency department
HIPAA: Health Insurance Portability and Accountability Act
ICF: informed consent form
SIM: Subscriber Identity Module
VA: Department of Veteran Affairs

## Data Availability

Datasets generated or analyzed during the study described by this protocol may be made available from the corresponding author on reasonable request.
